# Development of Regional Disparities in Alzheimer's Disease Mortality in the Slovak Republic from 1996 to 2015

**DOI:** 10.1155/2018/3149495

**Published:** 2018-10-11

**Authors:** Beáta Gavurová, Viliam Kováč, Dominika Jarčušková

**Affiliations:** ^1^Faculty of Economics, Technical University of Košice, Košice, Slovakia; ^2^Research and Innovation Centre Bioinformatics, University Science Park Technicom, Technical University of Košice, Košice, Slovakia; ^3^Department of Psychiatry, Louis Pasteur University Hospital, Košice, Slovakia; ^4^Faculty of Medicine, Pavol Jozef Šafárik University, Košice, Slovakia

## Abstract

Alzheimer's disease—subsequently as AD in the text—represents a chronic neurodegenerative disease discussed very often in the recent period. It involves the G30 diagnosis expressing exactly AD and also the F00 diagnosis epitomising dementia in AD. The Slovak Republic has a very various population in terms of the disparities of the population localisation. The analysis is executed on the basement of the standardised mortality rate. It is calculated for the individual districts of the Slovak Republic to get a detailed spatial view and for each year of the explored period from 1996 to 2015 to get a time development. It has a considerably rising tendency. Therefore, the regional disparities of the standardised mortality rate of AD are analysed from an angle of view of its similarity, by its measurement in a form of a Euclidean distance approach. The results of the analysis offer the heat maps as the distance matrices in a graphic form and the maps of the individual districts too. These outputs reveal a very heterogeneous structure of the standardised mortality rate. Another graphic outcome demonstrates a distribution of its values among the districts throughout the whole Slovak Republic for the whole observed period. The results offer a comparison among the districts of the Slovak Republic too. The highest values and also the lowest values are reached in the different districts for the both sexes. Even, one district reaches the opposite result for the individual sexes. The age structure of the deceased population on the G30 diagnosis is also executed and the extreme values from an angle of a view of the districts are picked up. There are evident high differentiations between the individual districts of the Slovak Republic. The conclusion section involves the several key points and the potential suggestions for further research.

## 1. Introduction

During the last decade the mortality rate of Alzheimer's disease has grown. This disease represents a degenerative brain disease and the most common cause of dementia. The person suffering from dementia has problems with their memory, language, thinking, and other cognitive skills which are also affected in a way which lowers this individual's ability to perform common everyday life activities.

The recently published study of Alzheimer's Association describes an impact of AD on public health, including incidence, prevalence, mortality rates, and health care costs as well as the impact on society and carers. Since the biomarkers could be important for both the diagnostic process and for the estimation of prevalence and incidence of the disease, it should be useful to do the investigation of their usage [1].

## 2. Literature Review

There are many scientific studies describing AD from the points of view analysed in this study.

The severity of AD, its consequences, and epidemic proportions related to the widespread social, health, and economic burden are often highlighted in the studies. The World Health Organization has described dementia and prevention of AD as a major public health priority. Both diseases have a wide range of the risk factors—including genetic, vascular, metabolic, and lifestyle aspects—that affect each other. The effectiveness of the preventive measures is determined by the age of the patient, which brings up a question of the correct timing. In terms of the complex multiple factor nature of AD as well as its long preclinical asymptomatic phase, the interventions which are simultaneously targeted at a number of the risk factors and the mechanisms of the disease whilst simultaneously applied at an early stage of the disease are most likely effective. Due to the processes of global aging, it is possible that the incidence of dementia will increase in the future. Delaying the onset of this disease will also greatly mitigate the social and economic burden [2].

The burden factors and management strategies for family caregivers of AD patients living in the community are a subject of examination. The results of the analysis have shown that higher cognitive, psychological, behavioural, and motor impairment in these patients is associated with an increasing level of burden and fear among caregivers who need to adopt the adequate management strategies and seek family and social support. The structure and volume of the disease costs are also strongly related to these aspects [3].

The costs of AD treatment are a subject of the study reported in a randomised controlled study. It stated the social burden of AD, comprising public, patient, and informal care costs around 20,000 € per year. The public sector costs 4,534 € per year. The portion of public expenditure is a national cash contribution at a level of 2,324 € per year and the prescriptions for the given medicine stand at 1402 € per year. A particular part of the expenditure is the cost of private workers. The AD burden reflects the structure of Italian welfare. The public spending is mainly allocated to medicines and cash benefits. From a point of view of the government, the benefit of these care arrangements is clear compared to the cost of the household care in the short term. However, if carers are not sufficiently supported, savings can soon be declared an offset by a higher risk of morbidity and mortality by the care provider due to high burden and stress [4].

Many studies address an impact of socioeconomic and genetic factors on AD. The aging-related diseases, including this one, have an epigenetic basis. The conclusion of the study is that even mild environmental stressors can result in epigenetic modifications. Among many different environmental factors influencing epigenome, nutrition is one of the most important areas [5].

Several research studies investigating etiological issues declare the significant differences between the different incidence rates of AD among male African Americans compared to female ones. Aggregation of this knowledge can be used to explore a potential burden of AD in American population in the future. These male inhabitants have an increased risk of disease incidence and prevalence compared to the female part of the population. The study looks for a cause for various biological, psychological and socioeconomic influences [6].

An attempt of a retrospective study to specify the profiles of patients with AD in the Commonwealth of Puerto Rico was done in 2010. It examines patients divided into two groups based on AD mortality, a low level of mortality and a high level of mortality. The other investigated parameters are a relationship between the comorbidities, the minorities, and the emergence of this disease in the Commonwealth of Puerto Rico. The differentiation profile of AD patients correlated with the differences in the socioeconomic status in the two regions of the country and suggests that a social status of population may contribute to the increased development risks of the disease. The study calls for potential follow-up studies to quantify the impact of the socioeconomic factors and a healthy lifestyle as the risk factors for AD [7].

Currently, around 47 million people live with dementia around the world. It is projected that this figure will increase to over 131 million by 2050 as the population ages. Dementia also has a huge economic impact. The total estimated cost of dementia is 818 billion USD worldwide. Although dementia can be diagnosed, the healthcare provided in countries is often fragmented, uncoordinated and does not respond to the needs of people with dementia, their families, and the caregivers. The health care for people suffering from dementia has to be interim, holistic, integrated, and adequate. The first point refers to treatment options, healthcare plans, and support requirements need to be monitored and revised when a situation develops and progresses. The second point means that there should be a treat with the whole person, not the individual conditions, the organs, or the systems and with regard to the unique context, the values, and the preferences of that person. The third point expresses a relationship between the healthcare providers, their level, and the health care system with the social system. The fourth point tries to ensure an adequate coverage of diagnostic services [8].

## 3. Materials and Methods

The analysis processes the data that are not public partially. The methodology is described in the next lines with the references to the original sources.

### 3.1. Data

The data comes from several sources. The data about mortality is provided by the National Health Information Center (Národné centrum zdravotníckych informácií) of the Slovak Republic. The population data is from the database of the Statistical Office of the Slovak Republic (Štatistický úrad Slovenskej republiky).

### 3.2. Methodology

The analysis is executed for the G30 diagnosis that represents Alzheimer's disease [9]. There is also an associated diagnosis marked F00, which epitomises dementia in Alzheimer's disease. Patients with AD can be classified using ether the G30 diagnosis describing AD commonly used by neurologist or the F00 diagnosis describing dementia in AD that is commonly used by psychiatrist [10]. But this minor diagnosis can be omitted because there is no recorded death on this diagnosis in the Slovak Republic for the whole observed period. The codes are applied as stated in the International Statistical Classification of Diseases and Related Health Problems, according to its tenth revision named ICD-10.

The standardised mortality rate is computed according to a sequence of the mathematical relations [11].

The basement is computed according to the succeeding relation:

(1) $$\begin{array}{c} SMR=\overset{g}{\underset{a=1}{\sum }}\left(\;\frac{M_{a}}{P_{a}}.{ESP}_{a}\;\right) \end{array}$$

where the individual variables mean that

- *SMR* is the standardised mortality rate;
- *g* is a number of age groups;
- *a* is the particular age group;
- *M*_*a*_ is a number of deaths in the a-th age group;
- *P*_*a*_ is a number of inhabitants in the a-th age group;
- *ESP*_*a*_ is a number of inhabitants in the a-th age group according to the European standard population [12].

The similarity of the individual administrative units is interpreted through the Euclidean distance [13].

Its computation is executed through the subsequent equation:

(2) $$\begin{array}{c} D\left(\;d_{1},d_{2}\;\right)=\sqrt{{\left({d_{1}}_{x}-{d_{2}}_{x}\right)}^{2}+{\left({d_{1}}_{y}-{d_{2}}_{y}\right)}^{2}} \end{array}$$

where the individual variables mean that

- *d*_1_ is the first district;
- *d*_2_ is the second district;
- *D*(*d*_1_, *d*_2_) is the mutual Euclidean distance of the d_1_ district and the d_2_ district;
- *d*_1__*x*_ is the x coordinate of the d_1_ district;
- *d*_2__*x*_ is the x coordinate of the d_2_ district;
- *d*_1__*y*_ is the y coordinate of the d_1_ district;
- *d*_2__*y*_ is the y coordinate of the d_2_ district.

To evaluate the districts during the whole observed period, variance of the Euclidean distances is calculated throughout this period. Subsequently, the first five districts with the lowest level of variance and the last five districts with the highest level of variance are selected to be displayed in the distant matrices. Such a selection is distinctive enough to create a picture about the regional disparities among the particular administrative units of the Slovak Republic in a field of their similarity in the standardised mortality rate due AD. The Euclidean distance is computed from the standardised mortality rate values meaning the mentioned coordinates entering its computation can serve to visualise the localisation of the particular districts in the two-dimensional area.

The regions of the explored territory are represented by the self-governing regions of the Slovak Republic that demonstrate the third level of the Nomenclature of Territorial Units for Statistics which serves as a basement for such spatial analysis in the documents of the Eurostat, the main statistical office of the European Union. The list of the self-governing regions is subsequent: BC: the Banská Bystrica Self-Governing Region; BL: the Bratislava Self-Governing Region; KI: the Košice Self-Governing Region, NI: the Nitra Self-Governing Region, PV: the Prešov Self-Governing Region, TA: the Trnava Self-Governing Region, TC: the Trnava Self-Governing Region, and ZI: the Žilina Self-Governing Region. The lower level is embodied by the fourth level of the Nomenclature of Territorial Units for Statistics and these administrative units are created as the districts in the Slovak Republic. They are numbered according to the district numbering system of the Slovak Republic made public in the appropriate promulgation of the Statistical Office of the Slovak Republic [14]. The list looks like as follows, whilst the districts are listed increasingly by their designation: 101, the Bratislava I District, 102, the Bratislava II District, 103, the Bratislava III District, 104, the Bratislava IV District, 105, the Bratislava V District, 106, the Malacky District, 107, the Pezinok District, 108, the Senec District, 201, the Dunajská Streda District, 202, the Galanta District, 203, the Hlohovec District, 204, the Piešťany District, 205, the Senica District, 206, the Skalica District, 207, the Trnava District, 301, the Bánovce nad Bebravou District, 302, the Ilava District, 303, the Myjava District, 304, the Nové Mesto nad Váhom District, 305, the Partizánske District, 306, the Považská Bystrica District, 307, the Prievidza District, 308, the Púchov District, 309, the Trenčín District, 401, the Komárno District, 402, the Levice District, 403, the Nitra District, 404, the Nové Zámky District, 405, the Šaľa District, 406, the Topoľčany District, 407, the Zlaté Moravce District, 501, the Bytča District, 502, the Čadca District, 503, the Dolný Kubín District, 504, the Kysucké Nové Mesto District, 505, the Liptovský Mikuláš District, 506, the Martin District, 507, the Námestovo District, 508, the Ružomberok District, 509, the Turčianske Teplice District, 510, the Tvrdošín District, 511, the Žilina District, 601, the Banská Bystrica District, 602, the Banská Štiavnica District, 603, the Brezno District, 604, the Detva District, 605, the Krupina District, 606, the Lučenec District, 607, the Poltár District, 608, the Revúca District, 609, the Rimavská Sobota District, 610, the Veľký Krtíš District, 611, the Zvolen District, 612, the Žarnovica District, 613, the Žiar nad Hronom District, 701, the Bardejov District, 702, the Humenné District, 703, the Kežmarok District, 704, the Levoča District, 705, the Medzilaborce District, 706, the Poprad District, 707, the Prešov District, 708, the Sabinov District, 709, the Snina District, 710, the Stará Ľubovňa District, 711, the Stropkov District, 712, the Svidník District, 713, the Vranov nad Topľou District, 801, the Gelnica District, 802, the Košice I District, 803, the Košice II District, 804, the Košice III District, 805, the Košice IV District, 806, the Košice-okolie District, 807, the Michalovce District, 808, the Rožňava District, 809, the Sobrance District, 810, the Spišská Nová Ves District, and 811, the Trebišov District. All the figures involved in the paper refer to this legend.

In a case of the same values reached by multiple districts, they are mentioned in an alphabetical order in the text of the paper.

The whole computation, the diagrams, and maps are produced by the R software environment. The maptools, rgdal, and shape packages serve to prepare the maps. The heat maps are generated by the gplots package and the remaining charts are produced by the ggplot2 package.

That is to note that all the figures and all the tables with the whole contents of the paper are elaborated by the authors.

## 4. Results and Discussion

Although this study focuses mainly on regional disparities, there are several other aspects on public health it describes including a comparison of the past and current situation. They are seen from numerous types of the statistical indicators.

### 4.1. Analysis of the Standardised Mortality Rate

**Figure 1:**
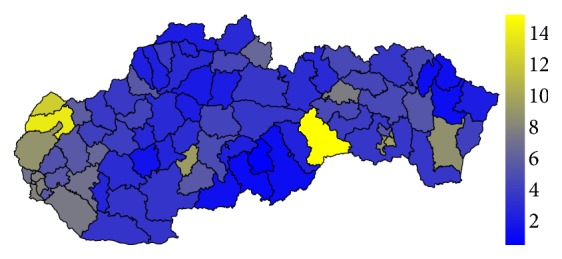
The average annual standardised mortality rate of the female sex in the individual districts of the Slovak Republic for the whole period.

**Figure 2:**
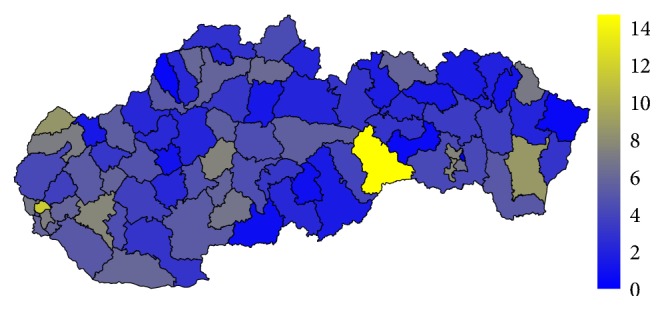
The average annual standardised mortality rate of the male sex in the individual districts of the Slovak Republic for the whole period.

**Figure 3:**
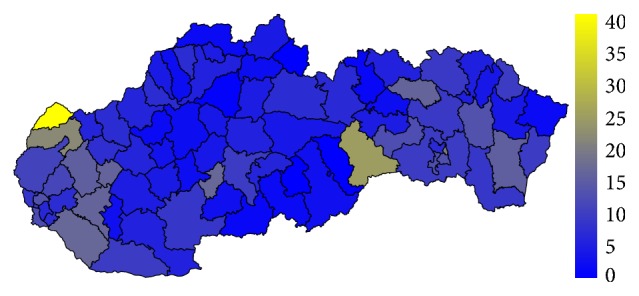
The average annual standardised mortality rate of the female sex in the individual districts of the Slovak Republic for the period from 2013 to 2015.

**Figure 4:**
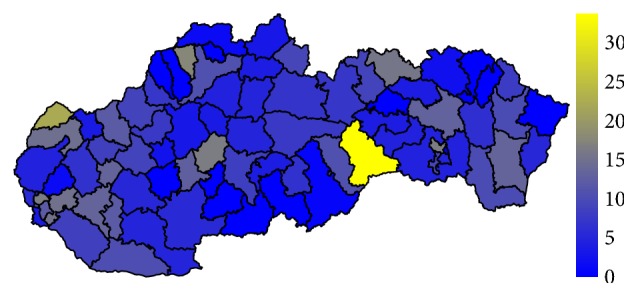
The average annual standardised mortality rate of the male sex in the individual districts of the Slovak Republic for the period from 2013 to 2015.

**Figure 5:**
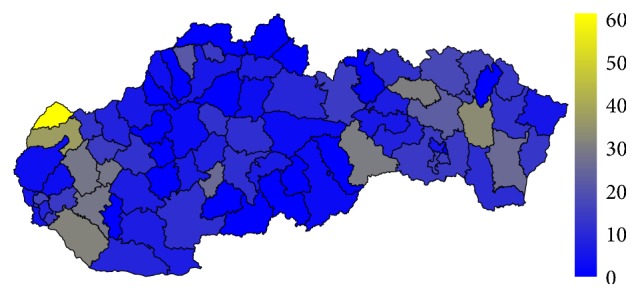
The standardised mortality rate of the female sex in the individual districts of the Slovak Republic in 2015.

**Figure 6:**
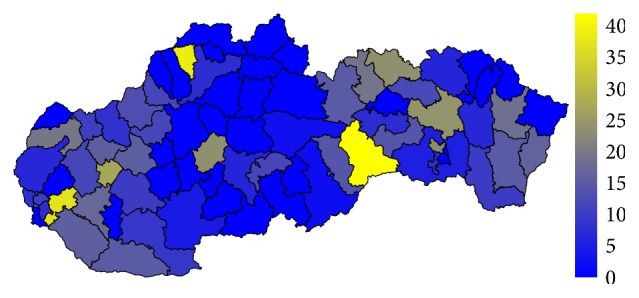
The standardised mortality rate of the male sex in the individual districts of the Slovak Republic in 2015.

Firstly, let us have a look at the situation in the field of standardised mortality rate on the G30 diagnosis. It is displayed for the two highest levels of administrative division of the territory of the Slovak Republic, for the self-governing regions as well as for the individual districts. The first pair of the tables—Tables 1 and 2—show the elementary statistic indicators, mean, variance, interquartile rang, minimum, and maximum of the values assigned to the particular self-governing regions of the Slovak Republic throughout the whole observed period. The involved numbers are rounded to the four decimal digits. The situation for the level of the districts is demonstrated in the maps in the second pair of tables—Tables 3 and 4—and on the successive figures. The first pair of the figures—Figures 1 and 2—shows an average annual value for the observed period from 1996 to 2015, the second pair—Figures 3 and 4—an average situation for the last three years of the explored period from 2013 to 2015, and finally, the third pair—Figures 5 and 6—a situation at the end of the observed period in 2015. The first map of the both pairs demonstrates a state of the female sex and the second one a state of the male sex.

In general, women are more susceptible to the G30 diagnosis than men. The figures for both sexes regarding the whole examined period from 1996 to 2015 are not very different. But, a closer look at the latest years reveal the disparities between the sexes. The highest average standardised mortality rate of the whole Slovak Republic is recorded in the Rožňava District at a level of 15.2056. A little cluster is formed by its two successors. The Senica District reached a value of 14.0328 with the Skalica District just behind with a value of 12.4342. All the other districts are below the two-digit threshold. On the other hand, the lowest standardised mortality rate below a threshold of 1 belongs to the Poltár District with a value of 0.4448.

The highest average standardised mortality rate of the whole Slovak Republic for the male sex is recorded in the Rožňava District at a level of 14.7885. The double-digit limit is also overstepped by the Bratislava III District with a value of 11.8596. Diversely, the lowest values below a threshold of one are kept by the Púchov District, the Snina District, the Gelnica District, the Veľký Krtíš District, and the Poltár District. Moreover, the Košice III District is the only administrative area where no person has ever died because of the G30 diagnosis. Undoubtedly, this fact is caused by the age structure of the district's population, which is very low, a lot of young people live there.

A comparison between the sexes comes out better for the male sex. The highest standardised mortality rate in the individual districts reaches a little lower number than for women. On an opposite side, there is only a sole district with below-a-one threshold for women, whilst there are 6 such districts for men with one at an absolute zero level.

Nowadays, the standardised mortality rate is much higher than it was a few years ago. It is up to ten times higher in the individual districts of the Slovak Republic in the present time.

In 2015, the standardised mortality rate is several times higher for both sexes. The most extreme value is recorded in the Skalica District for women where it reaches a level of 61.1749. For men, it is a 41.7374 value in the Rožňava District with the Bytča District and it is 39.2076 value and the Senec District at the level of 37.4846 in a hinge. There are also some districts with a zero level value, but these values are not well interpretable.

To avoid the potentially unsuitable result, a comparison between the average values of standardised mortality rate for the whole explored period and the average values of the last three years of this period is examined. Regarding only one year, there should be possibility that some districts fall to zero mortality, if no person dies on the G30 diagnosis. Nevertheless, a view in a form of the map displaying the individual districts of the Slovak Republic on the data from the last explored year 2015 is offered too.

A situation in the G30 diagnosis mortality has extremely changed over the observed period in both sexes. Also, there are some visible shifts from an angle of view of the individual districts. A better look at the current state of the standardised mortality rate offer a three-year average for the end of the observed period. Masterfully, the Skalica District keeps the worst position with a value of 41.3063, for the female sex. There is a huge gap behind it. It is followed by a triple of the districts with the values over 20 up to 25.5766, by the Rožňava District, the Košice III District, and the Senica District. The lowest values are assigned to the three districts with a zero level; these are the Poltár District, the Revúca District, and the Tvrdošín District. Also, the Martin District keeps its figure below a threshold of 1 at a level of 0.7862.

For the male sex, a situation in the time period is absolutely different in some cases. On the head, the Rožňava District with value of 33.5420 stands, followed by the Skalica District with a value of 22.4753. Subsequently, the other districts success after a gap. A zero level is kept by the thirteen districts even with another one below a threshold of 1. A group of these districts comprise the Banská Štiavnica District, the Košice III District, the Krupina District, the Kysucké Nové Mesto District, the Levoča District, the Lučenec District, the Považská Bystrica District, the Púchov District, the Snina District, the Stropkov District, the Svidník District, the Veľký Krtíš District, and the Zlaté Moravce District. Another one with low value is the Rimavská Sobota District with a 0.8100.

**Figure 7:**
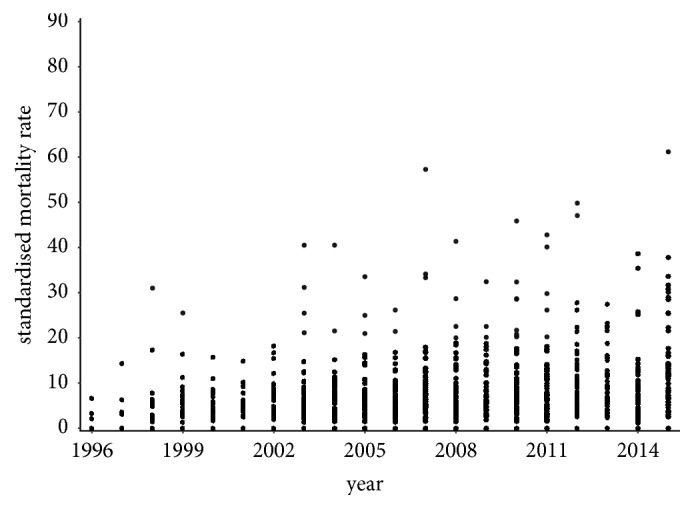
The standardised mortality rate of the individual districts of the Slovak Republic for the female sex throughout the whole period.

**Figure 8:**
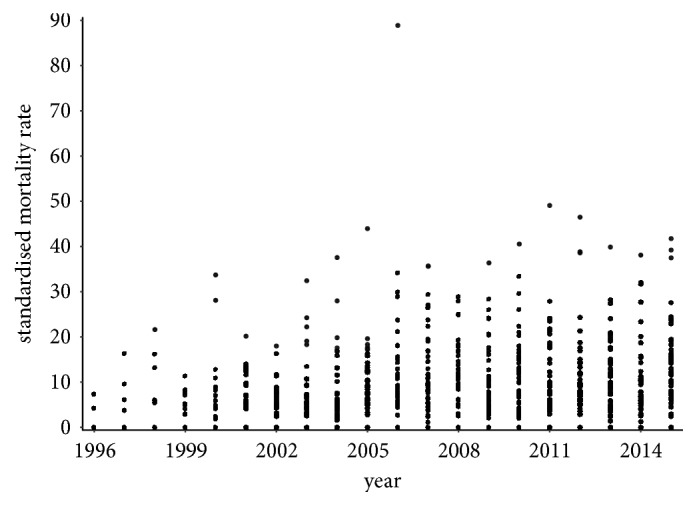
The standardised mortality rate of the individual districts of the Slovak Republic for the male sex throughout the whole period.

A visualisation of the standardised mortality rate of the individual districts is displayed in Figure 7 for the female population and in Figure 8 for the male population throughout the observed period.

### 4.2. Discrepancy in the Standardised Mortality Rate

The first five districts represent the administrative units with the lowest variance level of the standardised mortality rate reached throughout the whole observed period from 1996 to 2015 and the second five ones the highest variance. Variance itself demonstrates a presence of differentiations in the time period according to any time interval. In this case, it points to the rising standardised mortality rate; if variance increases its value, then mortality escalates in that period.

The lowest value of variance is recorded at level of 2.8446 by the Rimavská Sobota District in the case of the female population. The Poltár District follows with a value of 3.9577, the Lučenec District with a value of 4.3592, the Trenčín District with a value of 6.5846, and on the fifth place the Nitra District with a value of 6.6738. Differently, the highest value at level of 283.4436 is reached by the Rožňava District. The successive districts are the Košice III District with a value of 253.7411, the Skalica District with a value of 251.4497, the Banská Štiavnica District with a value of 204.4024 and on the fifth position the Senica District with a value of 196.6956. The lowest numbers are recorded partially in the neighbouring districts which could consider to create a cluster of the Lučenec District, the Poltár District and the Rimavská Sobota District. The opposite side could construct only a smaller cluster by the Senica District and the Skalica District. A median value of the female population for a whole set of the districts reaches a level of 26.1974 and is represented by the the Žarnovica District. A mean value stands at a level of 44.7024. The Bytča District bears the nearest value to this.

For the male population, the lowest value of variance at a zero level is reached by the Košice III District, then at a 4.8058 level by the Púchov District, at a 5.7071 level by the Snina District, at a 5.8131 level by the Rimavská Sobota District and finally, at a 6.2955 level by the Prievidza District. On the other hand, the highest level of variance is kept at a 435.9365 level by the Medzilaborce District. It is a very extreme value in terms of the whole data set. Then, the Rožňava District follows with the variance of 226.1461, the Krupina District with a 163.1513 level, the Bratislava III District with a 162.9006 level and finally, the fifth place is taken by the Hlohovec District with a 153.6871 level. The above-mentioned enumeration is partially heterogeneous. The given group of the districts involves mainly the areas from the poor regions. An exception is set by one of the Bratislava districts. As for a statistical comparison, a median value of the districts variance equals 34.1182 and is represented by the Levoča District. A mean value is quite different from the median and stands at 55.0866, which the Bratislava I District is the nearest one to. Again, there is to note that it is quite uncommon that one of the Bratislava districts bears a value that is the nearest one from the whole data set to the mean.

An interesting finding in the field of the regional disparities arises from the above-mentioned lines. Because the female population is more susceptible to death on the G30 diagnosis and it reaches lower variance of the standardised mortality rate than the male population at the same time, there are very high disparities among the districts of the Slovak Republic. Another point is that the Košice III District behaves most controversially. It keeps the zero standardised mortality rate for the male sex, but very high variance for the female sex.

### 4.3. Similarity of the Districts

The similarity of the individual districts of the Slovak Republic is explored through a Euclidean distance approach. It demonstrates how near are similar administrative units and vice versa, how far are dissimilar administrative units.

The distance matrices shown for the both sexes consist of ten rows and ten columns, because they comprise the same number of the most extreme districts. The first half involving the first five districts represents the administrative units with the lowest level of variance of the Euclidean distance values throughout the whole period. The rest of the distance matrix shows the five districts demonstrating the administrative units with the highest level of variance of the Euclidean distance; this means that these values have fluctuated most throughout the examined period.

**Figure 9:**
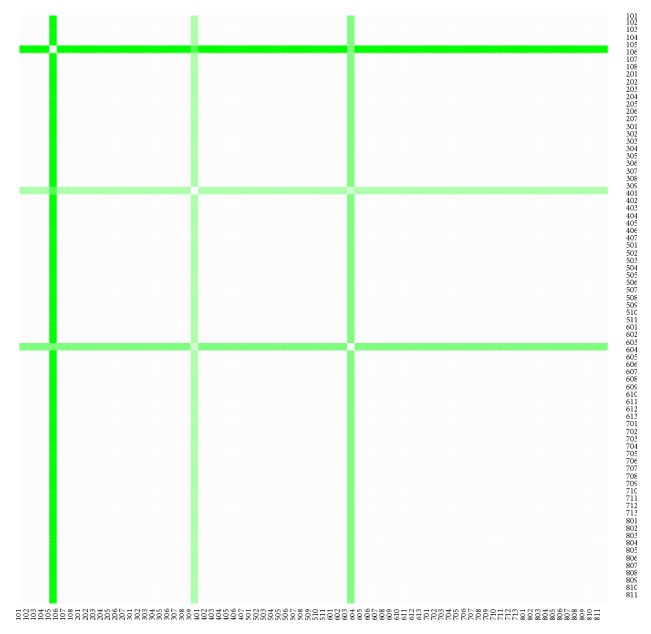
The distance matrix of the similarity of the districts for the female sex in 1996.

**Figure 10:**
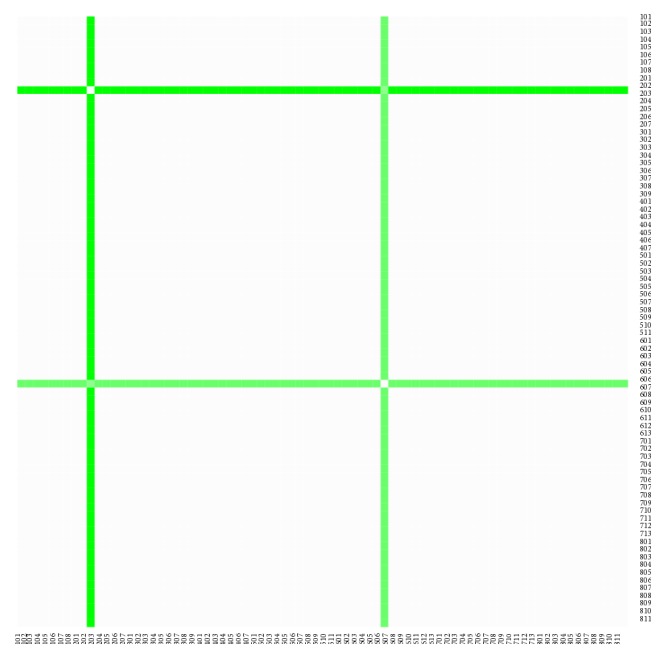
The distance matrix of the similarity of the districts for the male sex in 1996.

**Figure 11:**
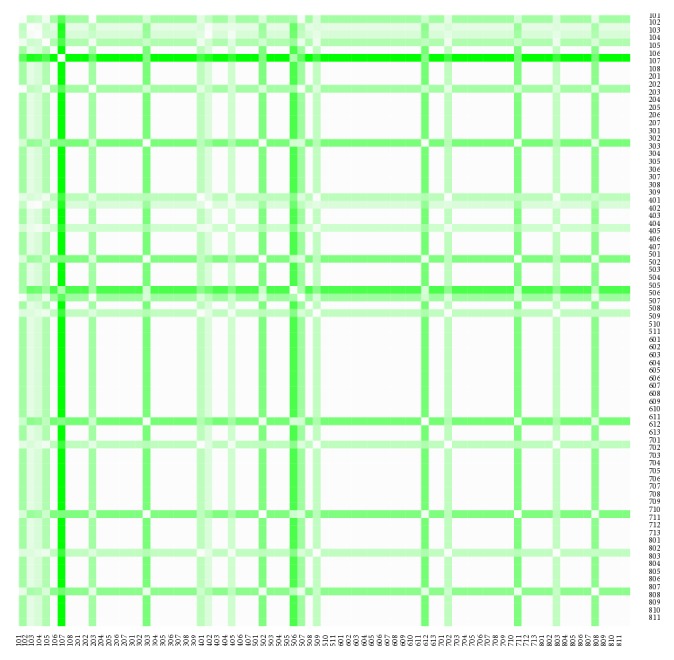
The distance matrix of the similarity of the districts for the female sex in 2000.

**Figure 12:**
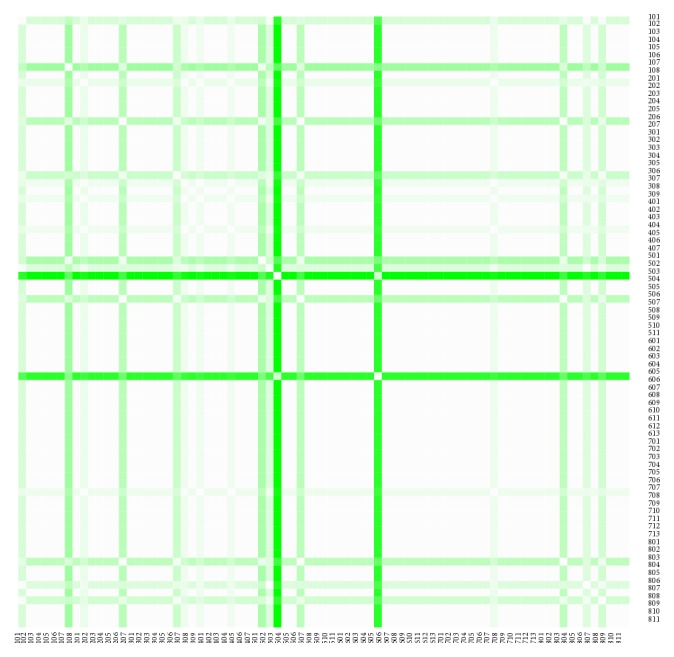
The distance matrix of the similarity of the districts for the male sex in 2000.

**Figure 13:**
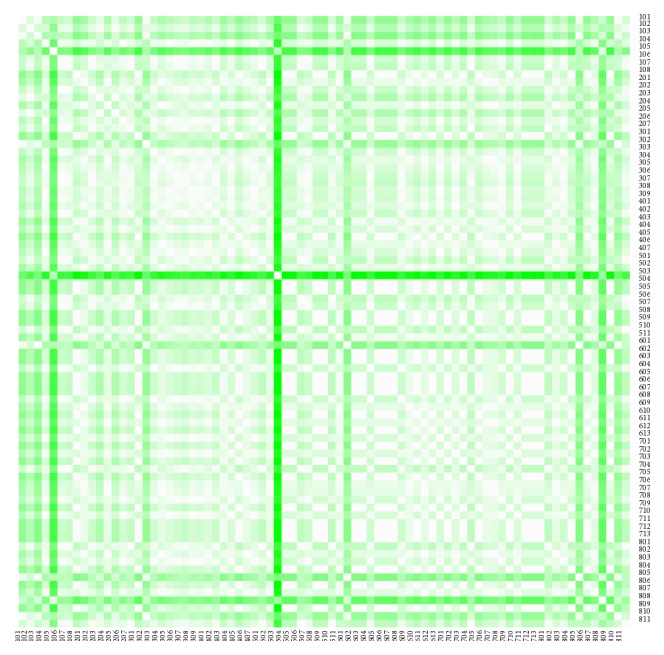
The distance matrix of the similarity of the districts for the female sex in 2005.

**Figure 14:**
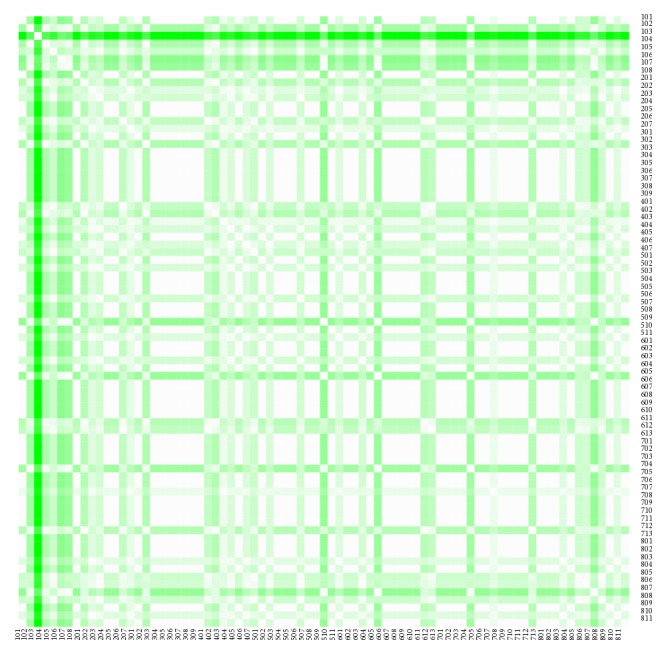
The distance matrix of the similarity of the districts for the male sex in 2005.

**Figure 15:**
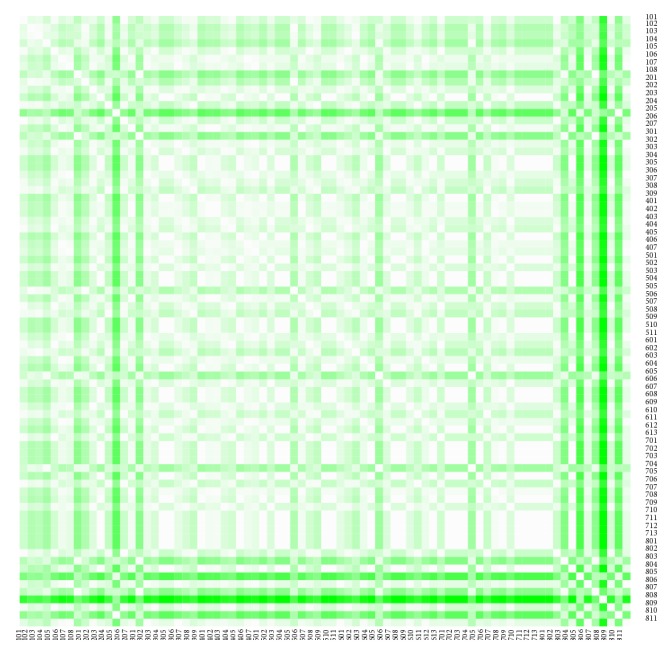
The distance matrix of the similarity of the districts for the female sex in 2010.

**Figure 16:**
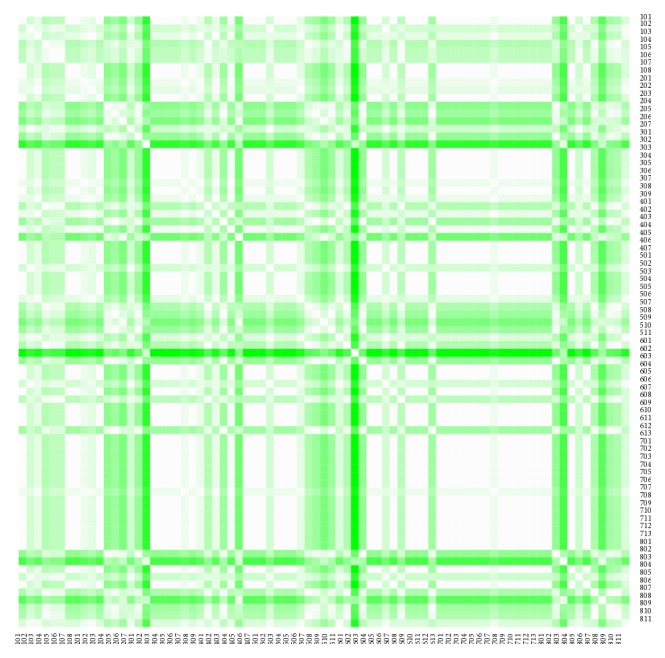
The distance matrix of the similarity of the districts for the male sex in 2010.

**Figure 17:**
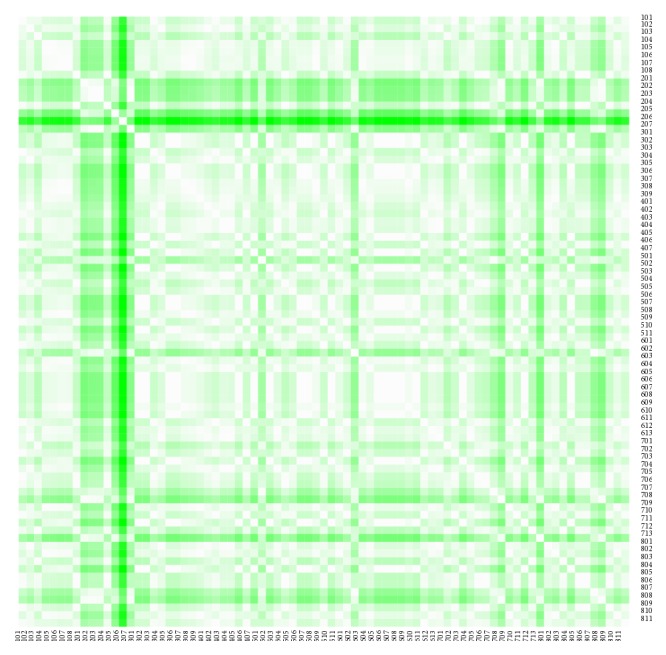
The distance matrix of the similarity of the districts for the female sex in 2015.

**Figure 18:**
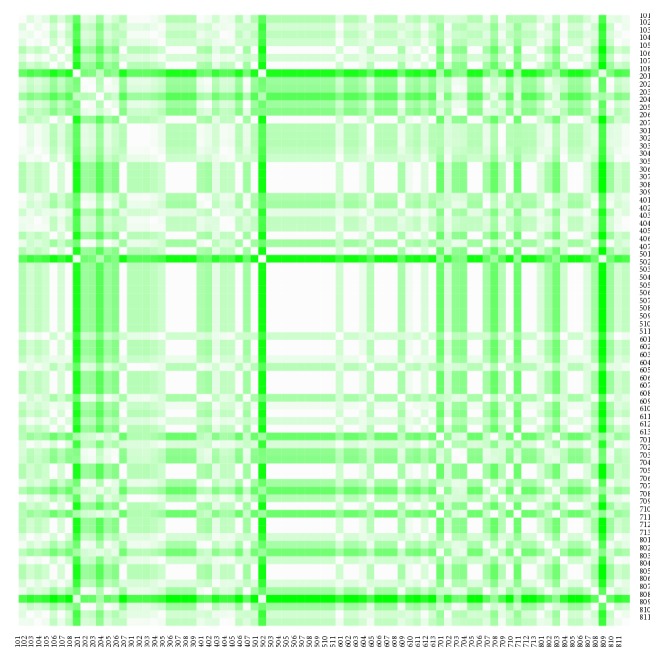
The distance matrix of the similarity of the districts for the male sex in 2015.

The heat maps demonstrate mutual similarity of all the districts of the Slovak Republic for both sexes separately. It is a graphic representation of the distance matrix. It is done for an approximately five-year interval with an exception of the first four-year interval. A situation at the beginning of the explored period in 1996 for the female sex is displayed in Figure 9, in 2000 in Figure 11, in 2005 in Figure 13, in 2010 in Figure 15, and at the end of the period in 2015 in Figure 17. For the male sex, a situation in 1996 is demonstrated in Figure 10, in 2000 in Figure 12, in 2005 in Figure 14, in 2010 in Figure 16, and finally, in 2015 in Figure 18. The colour shading legend is self-supporting, as white colour represents the highest similarity, whilst black colour represents the lowest similarity.

To demonstrate the ten most extreme districts from the mentioned angle of view, a look at the last observed year 2015 is presented. The both matrices are symmetric because of their characteristic.

The first distance matrix displayed as Table 1 shows the situation of the female sex. The lowest level of variance is kept by the Bratislava III District, the Dunajská Streda District, the Košice IV District, the Bratislava II District, and the Bratislava IV District. The highest variance level is reached by the Poltár District, the Lučenec District, the Rimavská Sobota District, the Veľký Krtíš District, and the Turčianske Teplice District. The lowest variance lies at a level of 0.0535, whilst the highest value is 0.1590.

The second distance matrix displayed as Table 2 demonstrates a situation for the male sex. The Skalica District, the Žilina District, the Bratislava II District, the Košice II District, and the Bratislava I District keep their variance values low, whilst the Košice III District, the Snina District, the Gelnica District, the Púchov District, the Veľký Krtíš District are on the opposite side of the spectrum. The lowest variance is at a level of 0.0449 and the highest variance at a level of 0.1197.

In the case of the female sex, there are very clearly visible the created clusters. The first one is consisted of the Bratislava districts—the Bratislava II District, the Bratislava III District, and the Bratislava IV District—together with the very near Dunajská Streda District. From an angle of view of the characteristics of these administrative units, another cluster can be created by the Bratislava districts with the Košice IV District according to their belongings to the large cities, although the Košice IV District does not bear absolutely urban characteristic rather than rural. The second cluster related to this outcome can be visible in the middle of the Slovak Republic consisted of the neighbouring districts with the highest variance of the Euclidean distance, by the Lučenec District, the Poltár District, the Rimavská Sobota District, and the Veľký Krtíš District.

The male sex brings up the more heterogeneous result. Whilst the lowest values create a geographical cluster of the two Bratislava districts, the Bratislava I District and the Bratislava II District, there is also visible a cluster of the large cities together with the Košice II District and the Žilina District. The highest values are borne by the totally heterogeneous districts. There is to note that the two Košice districts stand on the opposite sides of the spectrum.

Another interesting fact is caused by the Bratislava II District and the Veľký Krtíš District. The first one belongs to a group with the lowest variance level for the both sexes, whilst the second one belongs to a group with the highest variance level for the both sexes.

### 4.4. Age Structure of the Deceased Population

Age structure of the deceased population is quite various. It is unique for the both sexes. Generally, it can be said that the average age of the inhabitants who succumb to the G30 diagnosis has an increasing trend over the explored period.

At the beginning of the period in 1996, the average age of the female population for all the individual districts of the Slovak Republic reached a value of 72 years and it gradually rose up to 78.0102 years with the lowest extreme value of 62.75 years in 1997 and the highest extreme value of 81.0455 years in 2012. The male population has the little lower values; its time series begins with a value of 70 years in 1996 and ends with a value of 78.0984 years in 2015. The absolute minimum is reached at a level of 70 years in 1996 and on the other hand, the absolute maximum is kept at a level of 85.3333 in 1998. These mentioned extreme values are considerably touched by the fact that there is lower mortality on the G30 diagnosis during the first years of the explored period and hence, such outlier values are exposed.

From an angle of view of the individual districts throughout the whole period, the worst value for the female population is reached by the Púchov District at level of 71 years and the best value by the Myjava District at a level of 83.9444 years. The male population holds a minimum by a value of 69 years by the Turčianske Teplice District and a maximum by a value of 87.3333 years by the Medzilaborce District. There is to note that Košice III District has recorded no mortality on the G30 diagnosis. Another additional note is that this look should be considered in a synergy with a number of the deceased inhabitants in these particular districts.

## 5. Discussion

In this paper, a long-term perspective studying the development of the regional mortality disparities in the Slovak Republic is analysed. It follows these differences over the period of almost twenty years. This study has several results. The main outcome is that the female population has higher mortality rate compared to the male sex.

The previous studies have already found out that women are subject to a disproportionate burden from AD. One explanation of this phenomenon is that men may die of the competing causes of death earlier in life, so that only the most resilient men may survive to older age [15]. There are many factors which differentiate between the sexes, such as biological— the different sets of the chromosomes, the gonadal differences, and the hormonal differences—and cultural, an access to education and occupation [16]. These differences are not fully understood yet and their importance in developing AD with their mutual interactions were not described enough yet [17].

Further, the rate of cognitive decline with aging is also different between the sexes. Understanding the biology of sex differences in a cognitive function will not only provide insight into AD prevention, but is also integral to the development of personalised, gender-specific medicine [18].

Comparing the data recorded in 2015 to the data from the whole period from 1996 to 2015, it can be demonstrated that, with time, the burden of AD is rapidly expanding in the population. Roughly, it is up to ten times higher in the individual districts of the Slovak Republic, with biggest increase in the Rožňava District and the Skalica District. The increased standardised mortality rate is a subject that is very well described in literature. With a progressive increase in life expectancy, AD has become a rising public health issue. In 2013, the Global Burden of Disease Study found out that AD is one of the top fifty global causes of lost life years, which experienced a pronounced increase in the past few years [19]. It is estimated that in 2030 the disease will be the seventh highest cause of death in the high-income countries [20].

A few studies describe the trends of mortality from AD in the European Union. The overall standardised mortality rate increased from 28.18 to 45.19 throughout the period from 1994 to 2013. The entire European Union shows a statistically significant increase at a level of 5.6 % in the standardised mortality rate, with no identifiable join points. Mortality has risen over the last two decades in the 26 countries all over the European Union. In the ninetieth years of the twentieth century, the lowest mortality rates are observed in the mortality from the age-related diseases, especially in the eastern European countries such as Hungary, the Republic of Lithuania, the Republic of Slovenia, and Romania as well as the Slovak Republic. Due to an economic development and an improvement of social conditions over the last decades, life expectancy has increased. This might be a reason why the Slovak Republic and Romania had the largest increases in the mortality rates due to AD at level of 26 % and 22.7 %, respectively [21].

The other countries have also recorded increases in the AD mortality. The data from the United States of America for the period from 1999 to 2008 reveals that the standardised mortality rate rose from 45.3 to 50 [22]. Similarly, the data about the Canadian population from the period 2004 to 2011 found that the crude mortality rate for men and women increased from 10.1 to 11.5 and 24.4 to 25.4, respectively [23].

Since the highest average of the standardised mortality rate of the whole Slovak Republic for both sexes is found in the Rožňava District at a level of 14.7885, it can be theorised that an existence of certain factors causes it. It is most likely instigated by the genetic factors, such as familial AD that is an early onset AD. In this case, the individual has a heritable mutation that causes an onset of the clinical symptoms earlier in life, usually under the age of 65 [24]. The clinical course and neuropathology of familial AD and sporadic AD are highly similar [25].

This study has some limitations. Firstly, only the population of patients who died because of AD is studied. There might also be a big group of patients suffering from this disease that die because of different reasons. Thus, they are not included among them. Secondly, since this study is done using only the medical data, it is not understandable whether the patients are influenced by other risk factors for developing AD, such as ethnicity and family history of this disease, which can interfere with our results. Due to the same reason it is not possible to calculate the same data for the different group of patients—other nationalities and ethnicities—and compare them.

## 6. Conclusions

There is plenty of potential opportunities for future research which would further improve our understanding of AD in the Slovak Republic. It would be interesting to consider the extended analysis in the regional AD mortality trends in the neighbouring countries as well. Since the areas with the highest AD mortality rate are located on the boarders with the Czech Republic and Hungary, it might be attractive to study the regional disparities in this field in these states and to compare the results, because there is a potential incidence of familiar AD in the larger areas. Also, these facts may be caused by migration from the neighbouring countries or there might be some environmental factors. It would be interesting to genetically test the population from the Rožňava District, the Senica District and the Skalica District too, where the prevalence of the disease reaches the highest level as well as the Púchov District and the Turčianske Teplice district, where the population with AD is younger. These indicators might be underlain by genetic pathology. If this is true, an investigation about the type of pathology responsible for this disease should be undergone.

The findings of the studies could be of considerable relevance for the development of the healthcare system related to the AD treatment. It could help to begin the testing of the population from the regions, which are affected the most by AD before it develops in the population and help doctors direct the interventional treatment targeting the reduction of risk factors.

## Figures and Tables

**Table 1:** The elementary statistics for the female sex standardised mortality rate according to the regions.

district	mean	variance	interquartile range	minimum	maximum
BC	3.2749	6.5879	3.6270	0	9.6343
BL	7.3576	14.3302	4.9729	0.7886	13.6792
KI	6.9704	31.3492	10.3388	0	18.1614
NI	3.1449	4.2354	3.0049	0	6.8039
PV	3.6468	10.1908	2.9073	0	14.1870
TA	8.2255	54.4508	8.5693	0	32.9686
TC	3.7662	4.2085	3.7307	0.2355	8.3738
ZI	3.1940	4.2679	2.3250	0	6.8408

**Table 2:** The elementary statistics for the male sex standardised mortality rate according to the regions.

district	mean	variance	interquartile range	minimum	maximum
BC	4.0441	8.5754	4.1195	0	10.5534
BL	6.4249	22.0399	6.9005	0	15.1141
KI	5.6395	23.2500	6.4383	0	18.4039
NI	4.2782	7.6163	4.8157	0	9.5458
PV	2.7952	8.5466	3.4710	0	10.0283
TA	6.2156	19.2109	7.4943	0	15.2899
TC	2.5516	3.9695	2.2024	0	6.7924
ZI	3.8177	6.7308	2.7986	0	9.3630

**Table 3:** The distance matrix of the ten most extreme districts for the female sex in 2015.

district	103	201	805	102	104	509	610	609	606	607
103	0	1.3472	0.2170	0.2170	1.0410	0.4348	1.4963	1.2693	1.4963	1.4963
201	1.3472	0	1.5642	2.0897	2.3882	1.7820	2.8434	2.6165	2.8434	2.8434
805	0.2170	1.5642	0	0.5255	0.8240	0.2178	1.2793	1.0523	1.2793	1.2793
102	0.7425	2.0897	0.5255	0	0.2985	0.3077	0.7538	0.5268	0.7538	0.7538
104	1.0410	2.3882	0.8240	0.2985	0	0.6062	0.4552	0.2283	0.4552	0.4552
509	0.4348	1.7820	0.2178	0.3077	0.6062	0	1.0614	0.8345	1.0614	1.0614
610	1.4963	2.8434	1.2793	0.7538	0.4552	1.0614	0	0.2269	0	0
609	1.2693	2.6165	1.0523	0.5268	0.2283	0.8345	0.2269	0	0.2269	0.2269
606	1.4963	2.8434	1.2793	0.7538	0.4552	1.0614	0	0.2269	0	0
607	1.4963	2.8434	1.2793	0.7538	0.4552	1.0614	0	0.2269	0	0

**Table 4:** The distance matrix of the ten most extreme districts for the male sex in 2015.

district	206	511	102	803	101	610	308	801	709	804
206	0	0.7721	0.7324	0.7977	1.2541	0	0	1.4463	0	0
511	0.7721	0	0.0396	0.0257	0.4820	0.7721	0.7721	0.6742	0.7721	0.7721
102	0.7324	0.0396	0	0.0653	0.5216	0.7325	0.7325	0.7138	0.7325	0.7325
803	0.7977	0.0257	0.0653	0	0.4563	0.7978	0.7978	0.6485	0.7978	0.7978
101	1.2541	0.4820	0.5216	0.4563	0	1.2541	1.2541	0.1922	1.2541	1.2541
610	0	0.7721	0.7325	0.7978	1.2541	0	0	1.4463	0	0
308	0	0.7721	0.7325	0.7978	1.2541	0	0	1.4463	0	0
801	1.4463	0.6742	0.7138	0.6485	0.1922	1.4463	1.4463	0	1.4463	1.4463
709	0	0.7721	0.7325	0.7978	1.2541	0	0	1.4463	0	0
804	0	0.7721	0.7325	0.7978	1.2541	0	0	1.4463	0	0

## Data Availability

The data comes from the database of the National Health Information Center. There is a restriction that it is not publicly available because of its private characteristics. It includes information about the real inhabitants. The whole dataset is provided to the authors according to the mutual agreement about contractual cooperation between the provider and the authors.
